# Failure to account for psychiatric symptoms: Implications for the replicability and generalizability of psychological science?

**DOI:** 10.1017/S0033291725102237

**Published:** 2025-12-01

**Authors:** Eri Ichijo, Ka Shu Lee, Mirta Stantić, Isabel De Castro, Jennifer Murphy, Aikaterini Vafeiadou, Michael Banissy, Caroline Catmur, Geoffrey Bird

**Affiliations:** 1 Department of Experimental Psychology, University of Oxford, Oxford, UK; 2 Department of Psychology, Royal Holloway University of London, Egham, UK; 3 Department of Psychology, University of Southern Denmark: Syddansk Universitet, Odense, Denmark; 4 Department of Psychology, University of Surrey, Guildford, UK; 5 Department of Psychology, Goldsmiths University of London, London, UK; 6 School of Experimental Psychology, University of Bristol, Bristol, UK; 7 Department of Psychology, King’s College London, London, UK; 8 Centre for Research in Autism and Education, Institute of Education, University College London, London, UK

**Keywords:** anxiety, cognition, depression, psychiatric symptoms, reproducibility crisis

## Abstract

**Background:**

One of the challenges of psychological research is obtaining a sample representative of the general population. One largely overlooked participant characteristic is sub-clinical levels of psychiatric symptoms.

**Methods:**

A series of studies were conducted to assess (i) whether typical psychology study participants had more psychiatric symptoms than the general population, (ii) whether there are sub-groups defined by psychiatric symptoms within the no-diagnosis, no-medication participant pool, and (iii) whether sub-clinical levels of psychiatric symptoms have an effect on standard behavioral tasks. Five UK national datasets (*N* > 10,000) were compared to data from psychology study participants (Study 1: n = 872; Study 2: n = 43,094; Study 3: n = 267).

**Results:**

Psychology study participants showed significantly higher levels of anxiety and depression and lower well-being, according to four commonly used mental health measures (GHQ-12, PHQ-8, WEMWBS, and WHO-5). Five sub-groups within the psychology study participant group were identified based on symptom levels, ranging from none to significant psychiatric symptoms. These groupings predicted performance on tests of executive function, including the Stroop task and the n-back task, as well as measures of intelligence.

**Conclusions:**

This study demonstrates that standard psychology participant pools are unrepresentative and suggests that a failure to account for psychiatric symptoms when recruiting for any psychological study is likely to negatively impact the reproducibility and generalizability of psychological science.

The reproducibility crisis has gained attention in psychological science (Open Science Collaboration, 2015). Statistical power, selective reporting, and various aspects of between-study heterogeneity have been suggested as possible contributors to the crisis (Klein et al., 2018; Stanley et al., 2018). The present study focuses on the widespread use of opportunity samples in psychological science, which may contribute to between-study heterogeneity, impacting replicability of findings, and/or reduce the representativeness of study samples, impacting the validity of results. Psychological research typically relies on volunteers (often students), which almost inevitably results in unrepresentative sampling, including a skewed representation of demographic factors such as age, gender, and socioeconomic status (Lockwood & Van Den Bos, 2025; Nielsen et al., 2017). The same applies to a new wave of internet-mediated research using paid volunteer pools, which may have a higher proportion of casually employed individuals and those of lower socio-economic status than is representative of the general population. To account for some ways in which samples may be non-representative, information on a limited range of demographic variables (typically age and gender, sometimes ethnicity) is collected, and sometimes controlled for statistically.

Problematically for the representativeness of psychology volunteer samples, it is likely that the participant pool for psychological studies is not only unrepresentative in terms of demographic factors like age and socioeconomic status but also in the prevalence of sub-clinical levels of psychiatric symptoms. Participants are often recruited from populations who reportedly have increased levels of psychiatric symptoms, such as university students (Office for National Statistics, 2020) and those who are casually employed (Buckman et al., 2022).

This is especially problematic, as many psychological studies recruit individuals with no current or recent psychiatric diagnoses and those who are not currently taking or have recently taken psychotropic medication in an attempt to remove any effect of psychiatric conditions on variables of interest. This practice is followed by studies with no clinical focus, as well as studies that recruit control participants to compare with a clinical group. However, a lack of a diagnosis does not equate to an absence of symptoms, and while psychiatric diagnoses are binary (one either does or does not have a diagnosis), symptoms exist along a continuum (Crawford et al., 2011; Löwe et al., 2008). Psychiatric symptoms, even at sub-clinical levels, are known to affect cognitive abilities (Segal et al., 2015; Thoresen et al., 2016). It is therefore important that psychiatric symptoms are measured and controlled for, even in standard behavioral tasks with no ostensive clinical focus, to ensure that the reproducibility of studies is not compromised. This is especially true if levels of psychiatric symptoms are indeed higher (and not representative of the general population) in typical participant pools for psychological studies.

In Study 1, we investigated whether typical UK psychology study participants (PSPs) have more psychiatric symptoms compared to participants from national datasets, representative of the general population. It was hypothesized that compared to the national datasets, PSPs would have more psychiatric symptoms as measured by commonly used self-report questionnaires.

## Study 1

### Methods

#### Datasets

For UK national data, we obtained multi-stage stratified random samples via the UK Data Service (https://ukdataservice.ac.uk/). These datasets were selected based on five criteria: (i) large sample size (*N* > 10,000), (ii) data collected between 2010 and 2020, (iii) standardized measures of psychiatric symptoms and well-being, (iv) availability and quality of screening questions that allow for classifying participants as non-clinical, and (v) free data access. This resulted in five datasets for our analyses: The European Quality of Life Survey (EQoLS, 2011), the United Kingdom data from The European Health Interview Survey (EHIS, 2013), and Health Survey for England (HSE, 2016, 2018, and 2019). Missing questionnaire responses were imputed using the *missForest* package in R (Stekhoven & Buhlmann, 2012), provided that the questionnaires were at least 80% complete. Out-of-the-bag errors across datasets were negligible [2.55 × 10^−5^, 4.45 × 10^−4^], suggesting imputations were successful.

Eight hundred and seventy-two PSPs were compared to the national datasets. Participants completed an online questionnaire hosted on Gorilla (www.gorilla.sc; Anwyl-Irvine et al., 2020) between April and August 2020 (King’s College London [HR-19/20–17443, LRU-1920-17312]; University of Oxford [R70170/RE001]). Participants were recruited via social media, local advertisements, a psychology department participant recruitment system (www.sona-systems.com; SONA), and Prolific (www.prolific.com). Participants were awarded course credit if recruited through SONA, or entered into a prize draw upon completing the study.

To best replicate the standard practice of recruiting and/or identifying non-clinical participants, participants were first included if they (i) were aged between 18 to 64 years old (i.e. young to middle-aged adults; Franssen et al., 2020; Medley, 1980), (ii) had no psychiatric diagnoses, and (iii) did not use psychotropic medications at time of study administration or within the 6 months before. Age cut-offs were chosen based on age group data available from the national datasets. Critically, where information was available, participants were excluded if they (i) identified as neurodiverse, (ii) had other physical health issues (e.g. visual or hearing impairments), and (iii) did not provide demographic data. Finally, only UK-based participants were included to account for any national differences in mental health.

Table 1 summarizes the sample sizes for all datasets before and after applying the exclusion criteria (see Supplementary Table S1 for further details).

**Table 1.** An overview of study 1–3 data

Dataset	Year	*N*			Gender (%)			Median age group
		Initial	Final	Without ‘Other’	Male	Female	Other	
EQoLS	2011	142,435	3,316	3,316	43.9	56.1	-	40–44
EHIS	2013	20,161	9,179	9,179	43.2	56.8	-	45–49
HSE	2016	10,067	4,080	4,080	44.4	55.6	-	40–44
			4,081^a^	4,081	44.3	55.7	-	40–44
HSE	2018	10,250	4,004	4,004	44.3	55.7	-	40–44
HSE	2019	10,299	4,294	4,294	44.6	55.4	-	40–44
Study 1 PSP	2020	872	494	491	24.3	75.1	0.6	25–29
Study 2 PSP	2020	43,094	12,844	12,703	22.6	76.3	1.1	50–54
Study 3 PSP	2020–22	267	223	221	33.2	65.9	0.90	25–29

a HSE-2016 dataset differs in sample size depending on the questionnaire as participants did not have to complete both the GHQ-12 and WEMWBS.

*Note:* EQoLS: The European Quality of Life Survey; EHIS: the United Kingdom data from The European Health Interview Survey; HSE: Health Survey for England; PSP: Psychology Study Participants. National datasets used a binary option for gender (male, female), while the PSPs were provided with at least one more additional option of *‘Other’.* For ease of comparison, the *‘Other’* category was removed prior to analysis.

#### Measures and procedures

##### Questionnaire measures

The national datasets employed partially non-overlapping questionnaire measures. Included measures were the 5-item World Health Organization Well-Being Index (WHO-5; World Health Organization, 1998) in the EQoLS, the 8-item Patient Health Questionnaire (PHQ-8; Kroenke et al., 2001) in the EHIS, the General Health Questionnaire (GHQ-12; Goldberg & Williams, 1988) in the HSE 2016 and 2018, and the Warwick-Edinburgh Mental Wellbeing Scale (WEMWBS; Tennant et al., 2007) in the HSE 2016 and 2019.

The PSPs additionally completed the Beck Depression Inventory (BDI-II; Beck et al., 1996), Depression, Anxiety and Stress Scale (DASS-21; Lovibond & Lovibond, 1995), and Spielberger State/Trait Anxiety Inventory (STAI-1 and STAI-2; Spielberger et al., 1983). Internal consistencies were high across questionnaires (range: .837–.953).

Higher scores indicated greater symptom severity except for WEMWBS and WHO-5, where higher scores indicate better mental health. Participants were classified into categories (e.g. low, moderate, severe symptoms) according to each measure’s published cut-offs (see Supplementary Table S2). Below, these are referred to ‘questionnaire categories’.

#### Statistical analyses

All statistical analyses were performed using R (R Core Team, 2022) and RStudio (version 2022.12.0 + 353; RStudio Team, 2022), with an alpha threshold of *p* < .05 (two-tailed). All data were visualized using the *ggplot2* package (Wickham, 2016).

Multinomial logistic regressions were conducted using the *nnet* package (Venables & Ripley, 2002) to examine the relationship between questionnaire categories (i.e. scores falling between published ranges on the questionnaires indicating, for example, ‘none’, ‘moderate’, and ‘severe’ symptoms) and the predictor variables of data source (national datasets vs PSPs), gender, and age. As shown in Table 1, the gender ratio and age group of participants significantly differed between datasets; age group and gender were therefore added as fixed effects, as well as interaction terms with data source in analyses. Age was treated as a categorical variable with three levels: young (18–34 years old), middle aged (35–49 years old), and old (50–64 years old). Some questionnaire categories were collapsed to avoid small group sizes: ‘mild’ and ‘moderate’ categories were collapsed and labeled ‘moderate’ for GHQ-12 and PHQ-8, ‘moderately severe’ and ‘severe’ were collapsed and labeled ‘severe’ for PHQ-8, and ‘possible depression’ and ‘probable depression’ were combined and labeled ‘depression’ for the WEMWBS. Multinomial logistic regressions, rather than ordinal logistic regressions, were conducted as proportional odds assumptions were violated. Four models were fit and model fit compared. First was a simple model with data source, age category, and gender as fixed effects. Second was a model which added an interaction term between data source and gender to the simple model. Third was a model with all the terms from the simple model with an additional interaction term between data source and age. Fourth was a model with all the terms from the simple model, an interaction term between data source and gender, and an interaction term between data source and age. Model fit was assessed using Akaike Information Criterion (AIC) values and by conducting likelihood ratio tests. Higher level of well-being (e.g. no depression), the middle-aged age category, and male gender were used as references.

### Results

**Figure 1.**
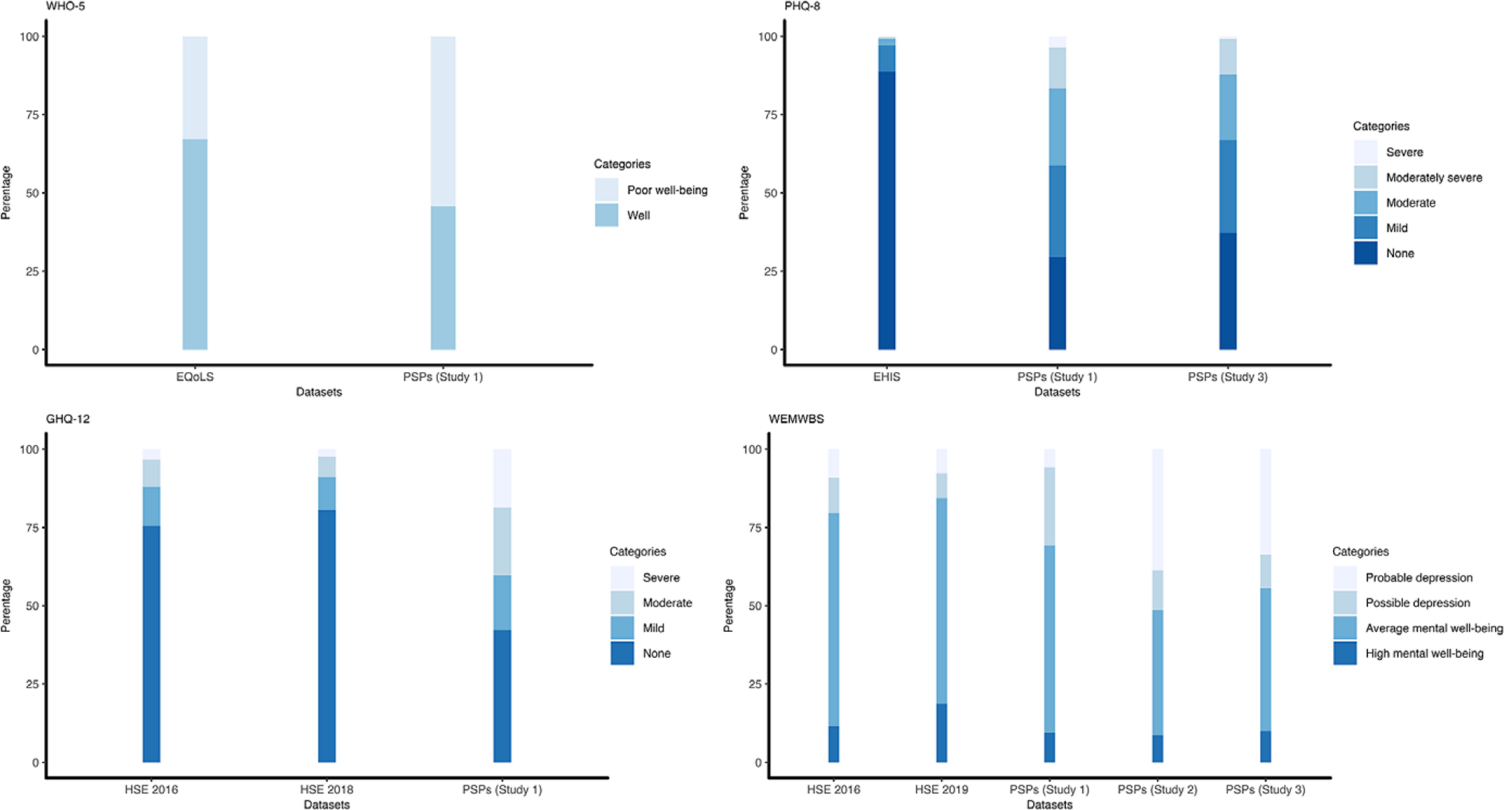
Proportions of participants classified into different symptom severity categories based on questionnaire cut-offs for Studies 1–3. *Note*: EQoLS: The European Quality of Life Survey; EHIS: the United Kingdom data from The European Health Interview Survey; HSE: Health Survey for England; PSP: Psychology Study Participants.

There were significant differences (*p* < .05) in the proportions of participants classified into different symptom severity categories based on questionnaire cut-offs between PSPs and the national datasets across all questionnaires, whereby PSPs had greater symptoms of poor mental health compared to the national datasets (Figure 1). Significant results including the data source are presented below (for full results, see Supplementary Materials – Study 1, Results).

45.8% of PSPs were categorized as ‘being well’ by the WHO-5, compared to 67.2% of individuals in the EQoLS. The model including an interaction term between data source and age had the best model fit (AIC = 4837.84). A participant from the EQoLS was 52.3% less likely to be categorized as having ‘poor well-being’ rather than ‘being well’ than a PSP ( 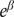 = 0.477 [0.291, 0.781], *p* = .003).

With respect to depression, 29.5% of PSPs were categorized as having ‘none’ on the PHQ-8, compared to 88.7% of individuals in the EHIS. The model including an interaction term between data source and age had the best model fit (AIC = 8014.94). A participant from the EHIS had significantly decreased odds (reductions of 89.5% and 97.2%) of being classified as having greater mental health symptoms (‘moderate’ and ‘severe’, respectively) than ‘none’ when compared with PSPs (*p* < .001; moderate: 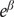 = 0.105 [0.062, 0.177]; severe: 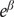 = 0.0282 [0.0120, 0.0660]). Additionally, being a young adult in the EHIS dataset decreased the odds of being classified as having ‘moderate’ depression rather than ‘none’ by 48.8% ( 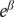 = 0.512 [.283, .926], *p* = .028), compared to a middle-aged adult PSPs.

When the level of psychiatric symptoms was assessed with the GHQ-12, 42.4% of PSPs were categorized as ‘none’ for psychological morbidity, compared to 75.5% of HSE-2016 and 80.5% of HSE-2018 respondents. The simple model provided the best fit to the data (AIC = 10904.36) where participants from the national datasets (HSE-2016, HSE-2018) were significantly less likely (67.0% and 74.9%, respectively) to be classified as having ‘moderate’ psychological morbidity rather than ‘none’ when compared with PSPs (*p* < .001; HSE-2016: 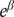 = 0.330 [0.265, 0.410]; HSE-2018: 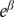 = 0.251 [0.201, 0.313]). A similar pattern was observed for the ‘severe’ category, where participants from the national datasets were significantly less likely (reductions of 87.4% for HSE-2016 and 91.9% for HSE-2019) to be categorized as having ‘severe’ levels of psychological morbidity than ‘none’ (*p* < .001; HSE-2016: 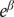 = 0.126 [0.0912, 0.174]; HSE-2018: 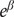 = 0.0812 [0.0576, 0.115]) compared to the PSPs.

Finally, 48.7% of the PSPs were categorized as having ‘average’ or ‘high mental well-being’ according to the WEMWBS, compared to national dataset figures of 79.6% for HSE-2016 and 84.3% for HSE-2019. The simple model provided the best fit to the data (AIC = 15269.14), where the odds of being categorized as ‘depressed’ rather than having ‘high mental well-being’ were significantly lower (65.3% for HSE-2016 and 83.5% for HSE-2019) for participants from national datasets (HSE-2016: 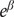 = 0.347 [0.243, 0.496], *p* < .001; HSE-2019: 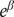 = 0.165 [0.116, 0.235], *p* < .001) than for PSPs. The odds of being categorized as having ‘average well-being’ rather than ‘high mental well-being’ was 47.3% higher for HSE-2016 participants than for PSPs ( 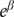 = 1.47 [1.03, 2.10], *p* = .0323).

### Discussion

Comparisons between PSPs and national datasets revealed a greater proportion of psychiatric symptoms among PSPs. These results indicate that PSPs are more likely to have higher symptom levels of various psychiatric conditions, or at least believe themselves to have higher levels of psychiatric symptoms, compared to the general population. However, data were collected for the PSPs during the COVID-19 pandemic, meaning that there exists an alternative explanation for the heightened levels of psychiatric symptoms seen in this group when compared with data collected before the pandemic (O’Connor et al., 2021).

## Study 2

### Introduction and methods

To remove any effect of the effect of the COVID-19 pandemic on symptoms of poor mental health, we compared data from 43,094 PSPs collected before the onset of the COVID-19 pandemic (Vafeiadou et al., 2022) and compared these data to the levels of psychiatric symptoms seen in the national datasets. In addition to the exclusion criteria used in Study 1, Study 2 responses completed after the beginning of the first national lockdown in the UK (23 March 2020) were excluded to provide a more accurate evaluation of pre-pandemic psychiatric symptoms in the general population. Participants completed the 7-item version of the WEMWBS (WEMWBS-7; Shah et al., 2021), hosted on Qualtrics (www.qualtrics.com; Qualtrics, 2020; Goldsmiths University [project reference 1521]). Participants were recruited and compensated as in Study 1. Table 1 summarizes the study sample sizes before and after applying the exclusion criteria (see Supplementary Table S1 for further details).

As in Study 1, multinominal regressions were used to assess the relationship between questionnaire categories and whether participant data was obtained from PSPs or national datasets.

### Results

Only significant results related to the comparison of PSPs to national datasets (Figure 1) are presented below. Full statistical analysis can be found in the Supplementary Materials – Study 2, Results.

69.4% of PSPs were categorized as having ‘average’ or ‘high mental well-being’ according to the WEMWBS, compared to 79.6% of respondents in the HSE-2016 and 84.3% in the HSE-2019 national datasets. The model including an interaction term between data source and gender, and an interaction term between data source and age had the best model fit (AIC = 37004.63). The odds of being categorized as ‘depressed’ rather than having ‘high mental well-being’ were 63.4% lower in HSE-2016, and 81.9% lower in HSE-2019 (*p* < .0001; HSE-2016: 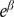 = 0.366 [0.272, 0.492]; HSE-2019: 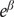 = 0.181 [0.138, 0.238]) than the PSPs included in Study 2. HSE-2019 respondents were also 49.8% less likely to be categorized as ‘average mental well-being’ than ‘high mental well-being’ compared to the PSPs ( 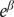 = 0.502 [0.398, 0.633], *p* < .0001). There were some interaction effects; female participants from HSE-2019 were 30.0% more likely to be categorized as having ‘depression’ than male PSPs ( 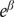 = 1.30 [1.00, 1.68], *p* = .0458), and older adults from HSE-2019 were 34.3% more likely to be categorized as having ‘depression’ than middle-aged adult PSPs ( 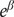 = 1.34 [1.01, 1.78], *p* = .0411).

### Discussion

Study 2 provided a conceptual replication of Study 1, showing that PSPs reported lower mental well-being than representative UK national datasets. This suggests that the elevated psychiatric symptoms found in PSPs in Study 1 compared to the national datasets cannot be explained as a pandemic effect.

## Study 3

### Introduction and methods

In Study 3, an additional independent sample of participants was recruited to further test whether elevated psychiatric symptoms are a generalizable feature of PSPs. A further aim was to assess whether participants who would be classed as non-clinical under typical criteria were truly a homogeneous non-clinical group with minimal psychiatric symptoms, or whether the participant pool consisted of various sub-groups characterized by distinct profiles of psychiatric symptoms. The final aim was to determine whether performance on standard cognitive tasks differs depending on the symptom profile of PSPs.

Two hundred and sixty-seven participants completed Study 3 between December 2020 and November 2022. Study 3 included the BDI-II, STAI-1, STAI-2, DASS-21, PHQ-8, WEMWBS, and three widely used behavioral tasks (Stroop, n-back, and matrix reasoning). Participants were recruited through the same means as Study 1, under the same ethical approval. Internal consistencies were again high across questionnaires for Study 3 (range: .865–.953). Table 1 summarizes the study sample sizes before and after applying the exclusion criteria (see Supplementary Table S1 for further details).

#### Behavioral tasks

Study 3 included the Stroop and n-back measures of executive function, and the matrix reasoning (MR) subtest of the Wechsler Abbreviated Scale of Intelligence (Second Edition, WASI-II; Wechsler, 2011). Accuracy and reaction time were collected for Stroop and n-back. For MR, the number of correct responses was transformed into T scores based on age using the WASI-II conversion tables.

Participants were removed prior to analyses if they scored below 80% for attention checks nested in the task, and if they answered incorrectly to the task-specific attention check question at the end of the task. The final sample included 220 participants for the Stroop task, 193 for the n-back task, and 222 for the MR task. Further task details can be found in the Supplementary Materials – Study 3, Methods.

#### Statistical analyses

Comparison of symptom levels between PSPs and national datasets were completed using multinomial regression as in Studies 1 and 2. To address the second aim (to determine whether the pool of ‘non-clinical’ PSPs consisted of sub-groups characterized by distinct profiles of psychiatric symptoms), a latent profile analysis (LPA) was conducted using the *mclust* package (Scrucca et al., 2016) across Studies 1 and 3 to identify any distinct psychiatric symptom profiles among PSPs in a multivariate manner. Bayesian Information Criterion (BIC) and integrated complete-data likelihood (ICL) were used to determine the best-fitting model. A Kruskal–Wallis test was employed to compare the median questionnaire scores between psychiatric symptom profiles, followed by Dunn’s test for pair-wise comparisons.

To address the third aim (to determine whether performance on standard cognitive tasks differs depending on the psychiatric profile of volunteers), polynomial trend analyses were conducted to assess whether there was a trend in task performance across psychiatric symptom profiles. Specifically, orthogonal polynomial coding was used to ascertain if there were polynomial effects on the outcome variables according to symptom severity. A contrast matrix was generated according to the number of psychiatric symptom profiles (i.e. *n* – 1). As behavioral task performance outcomes were not normally distributed, Kruskal–Wallis and Dunn’s test were used to determine whether psychiatric symptom profiles had an effect on task performance.

### Results

#### Prevalence of psychiatric symptoms

With respect to depression as assessed by the PHQ-8, 37.6% of PSPs were categorized as having ‘none’, compared to 88.7% of EHIS respondents. The model including an interaction term between data source and age had the best model fit (AIC = 7467.27). A participant from the EHIS national dataset has significantly reduced odds of having ‘moderate’ depression (by 85.1%), and ‘severe’ depression (by 93.4%) compared to PSPs (*p* < .0001; moderate: 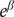 = 0.149 [0.0798, 0.277]; severe: 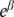 = 0.0659 [0.0187, 0.233]). Additionally, young adults from EHIS were 54.0% less likely than a middle-aged adult PSPs to be categorized as having ‘moderate’ depression than ‘none’ ( 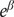 = 0.460 [0.220, 0.964], *p* = .0396).

When measured using WEMWBS, 56.2% of PSPs were categorized as having ‘average’ or ‘high mental well-being’ compared to 79.6% of respondents for HSE-2016 and 84.3% for HSE-2019. The simple model showed the best model fit (AIC = 14786.24), where the odds of being categorized as having ‘depression’ were 54.9% lower in HSE-2016, and 78.5% lower in HSE-2019 than for PSPs (HSE-2016: 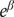 = 0.451 [0.279, 0.729], *p* = .00117; HSE-2019: 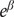 = 0.215 [0.133, 0.346], *p* < .0001). Questionnaire comparisons are visualized in Figure 1, and full statistical results can be found in the Supplementary Materials – Study 3, Results.

#### Are there distinct psychiatric symptom profiles in psychology study participants

**Figure 2.**
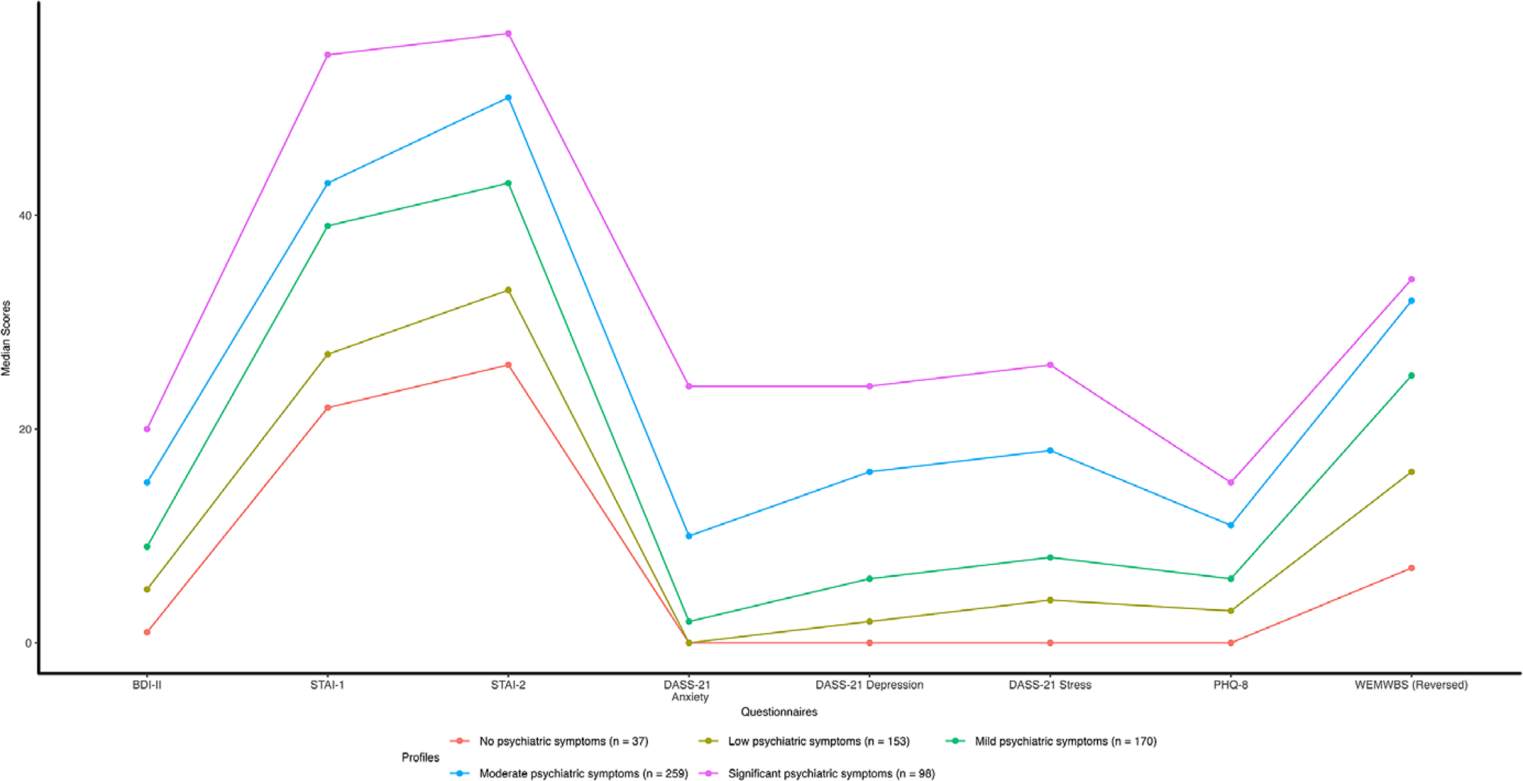
Psychiatric symptom profiles from ‘non-clinical’ psychology study participants from Studies 1 and 3 (N = 717). *Note*: Median scores of the BDI-II, STAI-1, STAI-2, subscales of anxiety, depression, and stress from DASS-21, PHQ-8, and WEMWBS were used to create psychiatric symptom profiles. WEMWBS scores are reverse scored as, unlike the other questionnaires, higher scores mean less symptom severity. Reverse scores were calculated by subtracting the scores from the highest score possible on WEMWBS, which is 70. Minimum scores for STAI-1 and STAI-2 are 20, whereas they are 0 for all other questionnaires.

LPA was used to identify distinct symptom profiles in a multivariate manner on data from PSPs from Studies 1 and 3. Figure 2 shows the five-cluster solution for 717 participants that best fit the data (‘VVE’ mixture, BIC = −36376.49, ICL = −36641.02), where psychiatric symptoms increase from ‘no’ to ‘significant psychiatric symptom’ profiles. The profiles map onto the questionnaire categories in the following manner: the ‘no’ (5.16%) and ‘low’ (21.34%) symptom profiles would be categorized across all questionnaires in the minimum category, the ‘mild’ profile (23.71%) in the minimal to moderate range on questionnaires, the ‘moderate’ profile (36.12%) in the mild to severe range, and the ‘significant’ symptom profile (13.67%) in the mild to extremely severe range across questionnaire categories.

Dunn’s multiple comparisons (see Supplementary Table S3) revealed significant differences between psychiatric symptom profiles across all questionnaires (*p* < .05) except for between ‘no’ and ‘low’ psychiatric symptoms profiles for STAI-2, DASS-21 anxiety and depression, and PHQ-8, and between ‘moderate’ and ‘significant’ psychiatric symptom profiles for WEMWBS.

#### Impact of psychiatric symptoms on behavioral task performance

##### Simple correlations

Significant correlations between behavioral task performance and self-report questionnaire scores were as follows: (i) Stroop effect accuracy with state and trait anxiety (STAI-1: *r* = .423, *p* < .05; STAI-2: *r* = .439, *p* < .05) and the anxiety subscale of the DASS-21 (*r* = .454, *p* < .05), (ii) Stroop median reaction time for incongruent trials with trait anxiety (STAI-2; *r* = −.428, *p* < .05) and the anxiety subscale of the DASS-21 (*r* = −.447, *p* < .05), and (iii) n-back median reaction time with trait anxiety (STAI-2; *r* = −.433, *p* < .05). All other correlations were non-significant (.000 ≤ 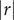 ≤ .419, *p* > .05).

##### Latent profile trend analysis

**Figure 3.**
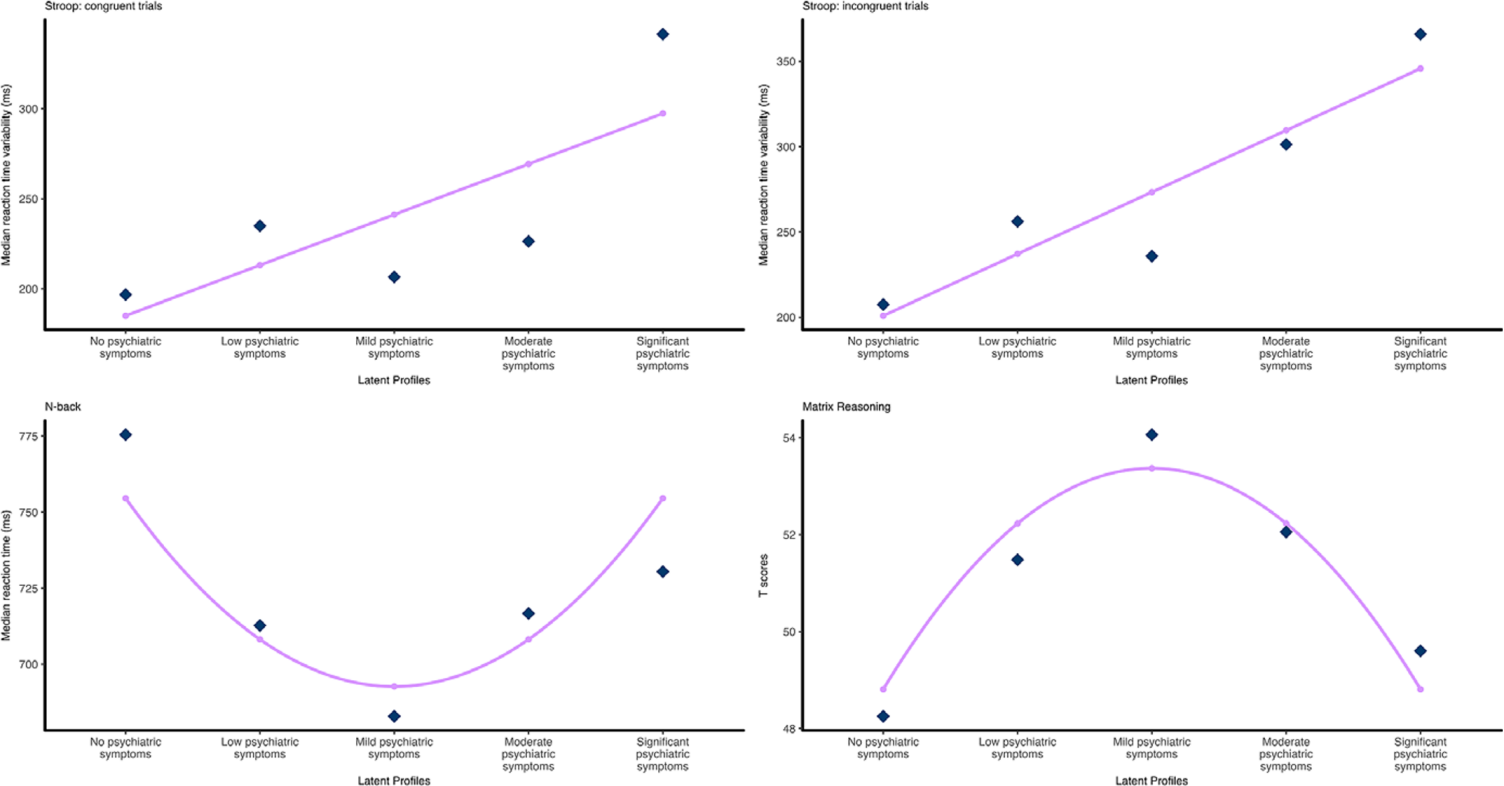
Relationships between psychiatric symptom profiles and behavioral task performance. *Note*: Dark diamond data points indicate actual mean values, and small dots indicate predicted values based on the best fitting polynomial contrast matrix from the trend analysis. Top: Stroop tasks, linear trend for reaction time variability for (left) congruent trials and (right) incongruent trials. Bottom: (left) quadratic trend for median reaction time for n-back task, (right) quadratic trend for Matrix Reasoning T-scores.

Orthogonal polynomial coding was used to test for any trends in behavioral task performance across the five psychiatric symptom profiles. Significant trends are described in Table 2 and illustrated in Figure 3. A full list of statistics can be found in Supplementary Tables S4 and S5.

**Table 2.** Descriptive statistics of behavioral task performance for Study 3 psychiatric symptom profiles

	*N*	Median	Psychiatric symptom profiles			
			No	Low	Mild	Moderate
Stroop congruent reaction time variability (ms)			χ^2^(4) = 2.23, 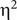 = − .008			
No	16	184	-			
Low	54	188	0.244	-		
Mild	48	181	0.300	.0876	-	
Moderate	74	193	0.649	0.612	0.497	-
Significant	28	221	1.16	1.26	1.16	0.832
Stroop incongruent reaction time variability (ms)			χ^2^(4) = 3.62, 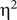 = − .002			
No	16	197	-			
Low	54	199	0.658	-		
Mild	48	230	0.823	0.253	-	
Moderate	74	221	1.31	0.974	0.670	-
Significant	28	240	1.59	1.33	1.09	0.610
N-back reaction time (ms)			χ^2^(4) = 5.75, 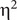 = .009			
No	15	743	-			
Low	49	682	−1.61	-		
Mild	42	661	−2.34	−1.09	-	
Moderate	61	685	−1.66	−0.0296	1.11	-
Significant	26	710	−1.20	0.346	1.25	0.383
Matrix reasoning (T-score)			χ^2^(4) = 6.92, 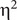 = .014			
No	16	51	-			
Low	54	52	1.56	-		
Mild	48	52	2.32	1.13	-	
Moderate	74	53	1.66	0.0738	−1.14	-
Significant	30	51.5	0.748	−0.932	−1.88	−1.04

*Note*: The last three columns represent Dunn’s multiple comparison Z-test statistics. * *p* < .05, ** *p* < .01, *** *p* < .001.

For the Stroop task, a linear trend was the best fitting model for reaction time variability for both congruent trials ( 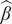 = 88.77, *p* = .013, adjusted *R*^2^ = .039), and incongruent trials ( 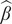 = 114.52, *p* = .033, adjusted *R*^2^ = .012). For the n-back task, median reaction time was shown to have a quadratic trend with a U-shape relationship between psychiatric symptom profiles ( 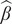 = 57.87, *p* = .026, adjusted *R*^2^ = .011). For the matrix reasoning data, T-scores were shown to have a quadratic trend with an inverse U-shape relationship between the psychiatric symptom profiles ( 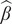 = −4.27, *p* = .008, adjusted *R*^2^ = .020).

### Discussion

Analysis of data from Study 3 revealed, like Studies 1 and 2, that PSPs have higher levels of psychiatric symptoms than reported in national datasets. Additionally, despite being recruited as a single group of healthy non-clinical study participants, an LPA of psychiatric symptoms revealed a five-cluster solution whereby the participants were divided into ‘no’, ‘low’, ‘mild’, ‘moderate’, and ‘significant psychiatric symptoms’ profiles. Results showed that the smallest group was the ‘no psychiatric symptoms’ profile (5.16%), and a significant proportion of participants were clustered into the ‘significant psychiatric symptoms’ profile (13.7%). In addition to highlighting once again that samples of ‘non-clinical’ volunteers for psychological studies are not without psychiatric symptoms, these data show the presence of five sub-groups in what is typically considered a homogeneous sample of ‘non-clinical’ volunteers. Subsequently, simple correlations and polynomial trend analysis were used to determine whether symptom profiles affected performance on standard tests of executive function and intelligence. In addition to several correlations between performance measures and psychiatric symptoms, significant linear trends for Stroop task reaction time variability for congruent and incongruent trials were seen, where the ‘no psychiatric symptoms’ profile had the smallest reaction time variability for both trial types. However, the ‘no psychiatric symptoms’ group had the longest median reaction time for the n-back task and also performed the worst on the matrix reasoning subtest of the WASI-II, which both showed a quadratic trend, such that the ‘mild psychiatric symptoms’ group performed the best on both the n-back and matrix reasoning. These findings support previous work demonstrating an effect of sub-clinical psychiatric symptoms on cognitive task performance (Segal et al., 2015; Thoresen et al., 2016).

## General discussion

The present study investigated the prevalence and effects of sub-clinical levels of psychiatric symptoms in what would typically be considered samples of non-clinical participants in psychology experiments. Across several studies, PSPs showed heightened levels of psychiatric symptoms compared to national datasets. In addition, the ‘non-clinical’ psychology participants had distinct psychiatric symptom profiles, ranging from ‘no’ to ‘significant’ psychiatric symptoms. These psychiatric symptom profiles explained significant variance on behavioral tasks widely used in the field of psychology. It should be noted, however, that it is possible that there are sub-groups even within the ‘no psychiatric symptoms’ profile, possibly comprised those who (i) truly do not have psychiatric symptoms and (ii) are not aware of their psychiatric symptoms, driven by factors such as dissociation (Lyssenko et al., 2018) or alexithymia (Nemiah, 1977).

What implications do these results have for the replicability and validity of psychological studies recruiting from typical participant pools? The first implication is that studies attempting to test ‘non-clinical individuals without a current or recent psychiatric diagnosis and not currently taking, or having recently taken psychotropic medicine’ are almost certainly still testing a substantial proportion of individuals with sub-clinical (and some with clinical) levels of psychiatric symptoms. If such symptoms are not measured, reported, and accounted for statistically, they will add to the unexplained variance in individual studies (reducing statistical power), and contribute to between-study heterogeneity and negatively impact reproducibility. Furthermore, the generalizability of results will be affected by the non-representativeness of participant samples with respect to psychiatric symptoms – potentially affecting test norms and the use of psychological data in legal, corporate, and policy settings.

The current study provides empirical evidence that standard participant pools have more psychiatric symptoms than the general population. One limitation of this study (in common with a number of psychological studies) is the unrepresentative age and gender ratio of the PSPs. Compared to the national datasets (median age group: 40–44), participants in Studies 1 and 3 were significantly younger (median age group: 25–29). It should be noted, however, that Study 2 did include a substantial proportion of older adults (median age group: 50–54), and yet it is still the case that a greater level of psychiatric symptoms was found compared to the national datasets. The PSPs also consisted of more female participants than the national datasets. Although limited age and gender effects were found, the effects were not consistent across questionnaires nor symptom severity. Indeed, the latest large-scale national study conducted in the UK by the Office of National Statistics (‘Quality of Life in the UK’, data collected October to December 2022, published 2023) showed no age effects on anxiety and mental well-being. The younger age of PSPs is an example of the general theme of this study; that typical psychological studies recruit from a pool of participants that is not representative of the general population.

The current study suggest that psychology participant pools have increased levels of psychiatric symptoms compared to the general population and that current screening measures to identify ‘non-clinical’ individuals do not serve to exclude individuals with psychiatric symptoms and do not result in a homogenous group. Consistent with previous research, even sub-clinical levels of symptoms impact performance on standard cognitive tasks, meaning that a failure to account for psychiatric symptoms likely contributes to reduced reproducibility in psychology and a lack of generalizability of findings.

## Supporting information

Ichijo et al. supplementary material Ichijo et al. supplementary material

## Supplementary material

The supplementary material for this article can be found at http://doi.org/10.1017/S0033291725102237.
