# Supplementary material for: Failure to account for psychiatric symptoms: Implications for the replicability and generalizability of psychological science?
Source: Psychol Med. 2025 Dec 1;55:e367. doi: 10.1017/S0033291725102237 (PMC13058620; doi:10.1017/S0033291725102237)
Supplement: Ichijo et al. supplementary material [file S0033291725102237sup001.docx]

**Supplementary Materials**

**General Methods**

***Participant Exclusion Process***

Participant exclusions were conducted prior to imputation of missing questionnaire responses (removing responses with less than 80% of questionnaire items completed), and after imputation (i.e., implementing the standard exclusion criteria for ‘non-clinical/control’ participants). Imputations were completed for all national datasets, and for psychology study participants in Study 2, which was a large-scale online dataset. Further details on the exclusion process can be found in Table 1.

**Table 1**

*Participant Exclusion Process.*

|  |  | National Datasets | | | | | | Psychology Study Participants | | |
| --- | --- | --- | --- | --- | --- | --- | --- | --- | --- | --- |
|  | | EQoLS | EHIS | HSE-2016 | | HSE-2018 | HSE-2019 | Study 1 | Study 2 | Study 3 |
|  |  |  |  | GHQ-12 | WEMWBS |  |  |  |  |  |
|  | Initial sample size | 142,435 | 20,161 | 10,067 |  | 10,250 | 10,299 | 872 | 43,094 | 267 |
| Pre-imputation | Non-UK | 136,360 | 0 | 0 | 0 | 0 | - | 200 | 19,070 | 37 |
|  | Post-lockdown responses^a^ | - | - | - | - | - | - | - | 283 | - |
|  | Proxy responses^b^ | - | 2,231 | - | - | - | - | - | - | - |
|  | Incomplete questionnaire  (less than 80% completed) | 1,027 | 84 | 2,767 | 3,000 | 2,644 | - | 173 | 54 | 4 |
|  | Incomplete demographics | 0 | 0 | 0 | 0 | 0 | - | - | 55 | - |
| Post-imputation exclusion | Outside age range | 1,479 | 6,785 | 2,360 | 2,127 | 2,588 | 15 | 0 | 7,894 | 0 |
|  | Presence of a mental health condition (e.g., depression) | - | 1,192 | 413 | 412 | 594 | 379 | 4 | 2,079 | 3 |
|  | Prescription of psychotropic medication (e.g., antidepressants) | - | - | 197 | 198 | 169 | 3 | 1 |  | 0 |
|  | A visit to a psychiatrist (past year) | - | 145 | - | - | - | - | - | - | - |
|  | Neurodiverse | - | - | - | - | - | - | - | 327 | - |
|  | Vision issues | - | 64 | 90 | 90 | 87 | - | - | 187 | - |
|  | Hearing issues | - | 193 | 56 | 54 | 71 | - | - | 256 | - |
|  | Very bad or bad general health | 253 | 288 | 104 | 105 | 93 | - | - | - | - |
|  | Final analysis sample | 3,316 | 9,179 | 4,080 | 4,081 | 4,004 | 408 | 494 | 12,844 | 223 |

*Note.* EQoLS: The European Quality of Life Survey; EHIS: the United Kingdom data from The European Health Interview Survey; HSE: Health Survey for England.

^a^Exclusion of post-lockdown responses is only applicable to Study 2.

^b^Examples for proxy responses include answering for other members of the household.

***Questionnaire Measures***

Table 2 summarises the various mental health/well-being measures used in the present study. While the inclusion of some of these measures was dependent on their availability in the national datasets, mental health measures widely used in psychological research and thus useful in evaluating mental health symptoms in our psychology study participants were selected.

**Table 2**

*Summary Table of Questionnaire Measures.*

| Questionnaire | Trait anxiety | Items | Score Range | Categories | |
| --- | --- | --- | --- | --- | --- |
|  |  |  |  | Severity | Scores |
| BDI-II | Depression | 21 | 0-63 | Minimal | 0-13 |
|  |  |  |  | Mild | 14-19 |
|  |  |  |  | Moderate | 20-28 |
|  |  |  |  | Severe | 29-63 |
| DASS-21 | Anxiety | 7 | 0-42 | Normal | 0-7 |
|  |  |  |  | Mild | 8-9 |
|  |  |  |  | Moderate | 10-14 |
|  |  |  |  | Severe | 15-19 |
|  |  |  |  | Extremely severe | 20+ |
|  | Depression | 7 | 0-42 | Normal | 0-9 |
|  |  |  |  | Mild | 10-13 |
|  |  |  |  | Moderate | 14-20 |
|  |  |  |  | Severe | 21-27 |
|  |  |  |  | Extremely severe | 28+ |
|  | Stress | 7 | 0-42 | Normal | 0-14 |
|  |  |  |  | Mild | 15-18 |
|  |  |  |  | Moderate | 19-25 |
|  |  |  |  | Severe | 26-33 |
|  |  |  |  | Extremely severe | 34+ |
| STAI-1 | State anxiety | 20 | 20-80 | Non-clinical anxiety | 20-39 |
| STAI-2 | Trait anxiety |  |  | Clinical anxiety | 40-80 |
| GHQ-12 | Psychological morbidity | 12 | 0-36 | None | 0-12 |
|  |  |  |  | Mild | 13-15 |
|  |  |  |  | Moderate | 16-20 |
|  |  |  |  | Severe | 21+ |
| PHQ-8 | Depression | 8 | 0-24 | None | 0-4 |
|  |  |  |  | Mild | 5-9 |
|  |  |  |  | Moderate | 10-14 |
|  |  |  |  | Moderately severe | 15-19 |
|  |  |  |  | Severe | 20+ |
| WEMWBS | Mental well-being | 14 | 14-70 | Probable depression | 0-40 |
|  |  |  |  | Possible depression | 41-44 |
|  |  |  |  | Average mental well-being | 45-59 |
|  |  |  |  | High mental well-being | 60+ |
| WEMWBS-7 |  | 7 | 7-35 | Probable depression | 0-17 |
|  |  |  |  | Possible depression | 18-20 |
|  |  |  |  | Average mental well-being | 21-27 |
|  |  |  |  | High mental well-being | 28+ |
| WHO-5 | Depression | 5 | 0-25 | Poor well-being | 0-13 |
|  |  |  |  | Well | 14+ |

**Study 1**

***Results***

For the WHO-5, in addition to the effect of the data source, there was an effect of age, such that older adults were 58.7% less likely to be classified as having “poor well-being” than being “well” compared to middle-aged adults ($e^{\beta}$ = 0.413 [0.173, 0.986], *p* = .046). Being female also increased the odds of being classified as having “poor well-being” than being “well” by 51.3% ($e^{\beta}$ =1.51 [1.32, 1.74], *p* < .001). No other terms were significant.

For the PHQ-8, there were additional significant effects of gender and age; for females, the odds of being classified as having “moderate” levels of depression increased by 27.7%, ($e^{\beta}$ = 1.28 [1.12, 1.46], *p* < .001), and younger adults were 100% more likely to be classified as having “moderate” levels of depression than “none” compared to middle-aged adults ($e^{\beta}$ = 2.00 [1.14, 3.51], *p* = .0151). No other terms were significant.

For the GHQ-12, there was also a significant effect of gender, where being female significantly increased the odds of being classified as having “moderate” or “severe” mental health symptoms rather than “none” by 20.9% and 46.2%, respectively (moderate: $e^{\beta}$ = 1.21 [1.08, 1.35], *p* < .001; severe: $e^{\beta}$ = 1.46 [1.15, 1.87], *p* = .002). There was also a significant effect of age limited to the moderate category, such that those in the old age category had a 15.0% reduction in the likelihood of being classified as having “moderate” mental health symptoms rather than “none” compared to those in the middle-aged category ($e^{\beta}$ = 0.850 [0.744, 0.970], *p* = .0163). No other terms were significant.

Finally, for the WEMWBS, there was also an effect of age; being young increased the odds of having “average mental well-being” by 17.8% and the odds of being “depressed” by 22.1% compared to middle-aged individuals (average mental well-being: $e^{\beta}$ = 1.18 [1.01, 1.38], *p* = .0416; depression: $e^{\beta}$ = 1.22 [1.01, 1.47], *p* = .0373). No other terms were significant.

**Study 2**

***Results***

In addition to the effect of the data source, there was an effect of age, and gender. First, being female decreased the odds of having “depression” by 16.2% ($e^{\beta}$ = 0.838 [0.718, 0.977], p = 0.0243). As for the effect of age, being an old adult reduced the odds of having poorer well-being than middle-aged adults (average mental well-being: $e^{\beta}$ = 0.778 [0.671, 0.901], *p* = 0.000847; depression: $e^{\beta}$ = 0.672 [0.575, 0.786], *p* < .0001), and being a young adult increased the odds of having “depression” by 53.1% ($e^{\beta}$ = 1.53 [1.19, 1.97], *p* = 0.000887). No other terms were significant.

**Study 3**

***Methods***

**Exclusion Criteria for Behavioural Tasks.** Exclusion criteria were applied across all three tasks. Any trials with a reaction time of less than 100ms were removed as these were likely accidental key-presses or responses where participants were not engaging with the task (Luce, 1991). An upper limit on reaction time was not set, to avoid potentially eliminating trials with lengthy reaction times as a result of any eﬀects of various mental health symptoms. As a result, median reaction times were used for analyses.

**Stroop Task.** Participants were presented with a colour word and were required to indicate whether the name of the colour and the font colour were the same or diﬀerent. The task consisted of 6 blocks of 24 Stroop trials (12 trials each of congruent and incongruent trials) and 2 attention check trials (a total of 144 Stroop trials and 12 attention check trials). Participants took self-timed breaks between blocks. Accuracy and reaction time were collected for congruent and incongruent trials separately. To quantify the Stroop eﬀect, the diﬀerence between incongruent and congruent trials was calculated for accuracy and reaction time, which was used for data analysis. Attention checks nested in the task asked participants to press the spacebar when they saw a screen with the words “Attention Check.” In the attention check questionnaire at the end of the study, participants were asked if they had to match the word to the shape, colour, or size of the letters.

**N-back Task.** A 2-back task was used, where participants were shown a series of letters and were instructed to indicate whether the letter shown on the screen, which remained for 2 seconds, was the same or diﬀerent as the letter shown two trials prior. The task consisted of 5 blocks of 30 trials and 1 attention check trial (a total of 150 n-back trials and 5 attention check trials). Accuracy and reaction time were collected. For data analysis, trials where participants did not produce a response were removed as it was not possible to determine whether it was due to lack of attention or their inability to give a response in the given time window (2 seconds). Attention checks nested in the task asked participants to press the spacebar when they saw a screen with the words “Attention Check,” which were presented before the self-timed break between blocks. In the attention check questionnaire at the end of the study, participants were asked if they had to match the letter that was one, two, or three letters before the one they were seeing.

**Matrix Reasoning Task.** The matrix reasoning (MR) sub-test from the Wechsler Abbreviated Scale of Intelligence (Second Edition, WASI-II; Wechsler, 2011) was used for this study to test nonverbal abstract problem solving. The number of correct responses was transformed into T scores based on age using the WASI-II conversion tables. No attention checks were created for this task.

***Results***

**Prevalence of Psychiatric Symptoms.** As seen in Studies 1 and 2, there were some effects of gender and being young. For the PHQ-8, there was an effect of gender, where being female increased the odds by 31.3% of being categorised as having “moderate” depression than “none” ($e^{\beta}$ = 1.31 [1.15, 1.50], *p* < .0001). There was also an effect of age, where being young increased the odds of being categorised as having “moderate” depression by 123% and “severe” depression by 282% (moderate: $e^{\beta}$ = 2.23 [1.09, 4.55], *p* = .0278; severe: $e^{\beta}$ = 3.82 [1.04, 14.0], *p* = .0436). No other terms were significant.

For the WEMWBS, there was an effect of age, where younger adults had poorer well-being than middle-aged adults (average mental well-being: $e^{\beta}$ = 1.18 [1.01, 1.39], *p* = .0378; depression: $e^{\beta}$ = 1.24 [1.02, 1.50], *p* = .0270), whereas being an old adult reduced the odds of being categorised as having “average mental well-being” over having “high mental well-being” by 17.5% ($e^{\beta}$ = 0.825 [0.7171, 0.949], *p* = .00730). No other terms were significant.

**Dunn’s Multiple Comparisons.** Table 3 shows descriptive statistics and full statistical results for Dunn’s multiple comparisons between all psychiatric symptom profiles.

**Simple Correlations for Self-Report Data.** As expected, all the self-report questionnaire scores were highly and significantly correlated with one another (.879 ≤ $\left| r \right|$ ≤ .987, .001 ≤ *p* ≤ .05). As for behavioural measures, Stroop median reaction time was correlated with age for congruent trials (*r* = .588, *p* < .01), and incongruent trials (*r* = .629, *p* < 0.01).

**Psychiatric Symptom Profile Trend Analysis.** Table 4 shows descriptive statistics and Table 5 shows statistical results for all variables used in the trend analysis for Stroop, n-back, and MR tasks. While no main effect of psychiatric symptom profile was found for any of the variables, some trends were found to be significant. For Stroop task, significant trends included reaction time variability for congruent (linear and cubic trends) and incongruent trials (linear trend). For n-back, it was a quadratic trend for median reaction time, and for MR, it was a quadratic trend for T-scores.

**Table 3**

Descriptive Statistics of Psychiatric Symptom Profiles From Psychology Study Participants Deemed To Be ‘Non-Clinical’ (N = 717).

|  |  |  |  | Psychiatric Symptom Profiles | | | |
| --- | --- | --- | --- | --- | --- | --- | --- |
|  | Median | Min | Max | No | Low | Mild | Moderate |
| BDI-II | | | | 𝜒^2^(4) = 390.06***, η^2^ = .542 | | | |
| No | 1 | 0 | 4 | - |  |  |  |
| Low | 5 | 0 | 15 | 3.14* | - |  |  |
| Mild | 9 | 0 | 19 | 6.44*** | 5.32*** | - |  |
| Moderate | 15 | 1 | 49 | 11.57*** | 14.30*** | 8.76*** | - |
| Significant | 20 | 0 | 49 | 12.59*** | 14.33*** | 9.94*** | 3.34** |
| STAI-1 | | | | 𝜒^2^(4) = 350.27***, η^2^ = .486 | | | |
| No | 22 | 20 | 25 | - |  |  |  |
| Low | 27 | 20 | 40 | 3.14* | - |  |  |
| Mild | 39 | 21 | 57 | 8.69*** | 8.98*** | - |  |
| Moderate | 43 | 20 | 71 | 10.85*** | 13.05*** | 3.34** | - |
| Significant | 55 | 22 | 80 | 12.82*** | 14.68*** | 7.08*** | 4.79*** |
| STAI-2 | | | | 𝜒^2^(4) = 462.13***, η^2^ = .643 | | | |
| No | 26 | 23 | 30 | - |  |  |  |
| Low | 33 | 23 | 41 | 2.52 | - |  |  |
| Mild | 43 | 28 | 57 | 8.14*** | 9.11*** | - |  |
| Moderate | 51 | 25 | 69 | 12.07*** | 16.27*** | 6.52*** | - |
| Significant | 57 | 37 | 77 | 13.45*** | 16.49*** | 8.82*** | 4.00*** |
| DASS-21 Anxiety | | | | 𝜒^2^(4) = 472.24***, η^2^ = .658 | | | |
| No | 0 | 0 | 2 | - |  |  |  |
| Low | 0 | 0 | 10 | 1.65 | - |  |  |
| Mild | 2 | 0 | 10 | 3.48** | 2.95* | - |  |
| Moderate | 10 | 0 | 20 | 8.99*** | 12.53*** | 9.62*** | - |
| Significant | 24 | 16 | 42 | 13.72*** | 18.12*** | 15.90*** | 9.00*** |
| DASS-21 Depression | | | | 𝜒^2^(4) = 435.16***, η^2^ = .606 | | | |
| No | 0 | 0 | 6 | - |  |  |  |
| Low | 2 | 0 | 14 | 2.43 | - |  |  |
| Mild | 6 | 0 | 18 | 5.44*** | 4.86*** | - |  |
| Moderate | 16 | 0 | 42 | 11.01*** | 14.61*** | 9.60*** | - |
| Significant | 24 | 2 | 42 | 13.01*** | 15.96*** | 12.01*** | 4.85*** |
| DASS-21 Stress | | | | 𝜒^2^(4) = 412.64***, η^2^ = .574 | | | |
| No | 0 | 0 | 2 | - |  |  |  |
| Low | 4 | 0 | 16 | 3.52** | - |  |  |
| Mild | 8 | 0 | 22 | 5.98*** | 3.95*** | - |  |
| Moderate | 18 | 0 | 42 | 11.23*** | 13.03*** | 9.00*** | - |
| Significant | 26 | 6 | 42 | 13.76*** | 15.54*** | 12.38*** | 5.75*** |
| PHQ-8 | | | | 𝜒^2^(4) = 469.08***, η^2^ = .653 | | | |
| No | 0 | 0 | 3 | - |  |  |  |
| Low | 3 | 0 | 9 | 2.77 | - |  |  |
| Mild | 6 | 0 | 12 | 6.27*** | 5.66*** | - |  |
| Moderate | 11 | 1 | 23 | 11.96*** | 15.65*** | 9.78*** | - |
| Significant | 15 | 2 | 24 | 13.47*** | 16.18*** | 11.53*** | 4.19*** |
| WEMWBS | | | | 𝜒^2^(4) = 371.59***, η^2^ = .516 | | | |
| No | 63 | 58 | 70 | - |  |  |  |
| Low | 54 | 24 | 70 | -3.34*** | - |  |  |
| Mild | 45 | 28 | 66 | -8.26*** | -7.96*** | - |  |
| Moderate | 38 | 14 | 68 | -12.07*** | -14.79*** | -6.29*** | - |
| Significant | 36 | 14 | 60 | 12.21*** | -13.48*** | -6.76*** | -1.99 |

*Note.* Median, minimum, and maximum scores per questionnaire are shown for each psychiatric symptom profile. The last three columns represent adjusted Dunn’s multiple comparisons. * *p* < .05, ** *p* < .01, *** *p* < .001.

**Table 4**

*Descriptive Statistics of Behavioural Task Performance for Psychiatric Symptom Profiles.*

|  |  |  | Psychiatric Symptom Profiles | | | |  |  |  | Psychiatric Symptom Profiles | | | |
| --- | --- | --- | --- | --- | --- | --- | --- | --- | --- | --- | --- | --- | --- |
|  | *N* | Median | No | Low | Mild | Moderate |  | *N* | Median | No | Low | Mild | Moderate |
| Stroop Task | | | | | | | | | | | | | |
| Accuracy: congruent | | | 𝜒^2^(4) = 4.73, $\eta$^2^ = .0003 | | | | Accuracy: incongruent | | | 𝜒^2^(4) = 5.71, $\eta$^2^ = .0008 | | | |
| No | 16 | 98.6 | - |  |  |  | No | 16 | 99.3 | - |  |  |  |
| Low | 54 | 97.2 | -1.01 | - |  |  | Low | 54 | 98.6 | -1.52 | - |  |  |
| Mild | 48 | 96.5 | -1.72 | -1.06 | - |  | Mild | 48 | 98.6 | -2.13 | -0.915 | - |  |
| Moderate | 74 | 96.5 | -1.57 | -0.808 | 0.351 | - | Moderate | 74 | 98.6 | -1.75 | -0.283 | 0.705 | - |
| Significant | 28 | 95.8 | -1.87 | -0.368 | -0.368 | -0.688 | Significant | 28 | 97.9 | -2.15 | -1.04 | -0.253 | -0.86 |
| Accuracy: difference | | | 𝜒^2^(4) = 0.150, $\eta$^2^ = -.0179 | | | | Median RT: congruent (ms) | | | 𝜒^2^(4) = 1.85, $\eta$^2^ = -.0100 | | | |
| No | 16 | 1.39 | - |  |  |  | No | 16 | 715 | - |  |  |  |
| Low | 54 | 1.39 | -0.313 | - |  |  | Low | 54 | 687 | -1.07 | - |  |  |
| Mild | 48 | 1.39 | -0.224 | 0.123 | - |  | Mild | 48 | 677 | -1.27 | -0.324 | - |  |
| Moderate | 74 | 1.39 | -0.233 | 0.14 | -0.003 | - | Moderate | 74 | 685 | -0.89 | 0.323 | 0.659 | - |
| Significant | 28 | 1.39 | -0.064 | 0.297 | 0.188 | 0.198 | Significant | 28 | 674 | -1.09 | -0.168 | 0.105 | -0.437 |
| Median RT: incongruent (ms) | | | 𝜒^2^(4) = 1.59, $\eta$^2^ = -.0112 | | | | Median RT: difference (ms) | | | 𝜒^2^(4) = 4.82, $\eta$^2^ = .00383 | | | |
| No | 16 | 760 | - |  |  |  | No | 16 | 34.4 | - |  |  |  |
| Low | 54 | 744 | -0.510 | - |  |  | Low | 54 | 54.8 | 0.564 | - |  |  |
| Mild | 48 | 733 | -0.999 | -0.723 | - |  | Mild | 48 | 38.4 | -0.336 | -1.30 | - |  |
| Moderate | 74 | 725 | -0.749 | -0.343 | 0.443 | - | Moderate | 74 | 46.2 | 0.189 | -0.605 | 0.804 | - |
| Significant | 28 | 730 | -1.03 | -0.764 | -0.145 | -0.525 | Significant | 28 | 31.9 | -1.01 | -2.04 | -0.920 | -1.66 |
| RT variability: congruent (ms) | | | 𝜒^2^(4) = 2.23, $\eta$^2^ = -.008 | | | | RT variability: incongruent (ms) | | | 𝜒^2^(4) = 3.62, $\eta$^2^ = -.002 | | | |
| No | 16 | 184 | - |  |  |  | No | 16 | 197 | - |  |  |  |
| Low | 54 | 188 | 0.244 | - |  |  | Low | 54 | 199 | 0.658 | - |  |  |
| Mild | 48 | 181 | 0.300 | 0.0876 | - |  | Mild | 48 | 230 | 0.823 | 0.253 | - |  |
| Moderate | 74 | 193 | 0.649 | 0.612 | 0.497 | - | Moderate | 74 | 221 | 1.31 | 0.974 | 0.67 | - |
| Significant | 28 | 221 | 1.16 | 1.26 | 1.16 | 0.832 | Significant | 28 | 240 | 1.59 | 1.33 | 1.09 | 0.61 |
| RT variability: difference (ms) | | | 𝜒^2^(4) = 2.60, $\eta$^2^ = .00649 | | | |  |  |  |  |  |  |  |
| No | 16 | -1.24 | - |  |  |  |  |  |  |  |  |  |  |
| Low | 54 | 26.1 | 1.24 | - |  |  |  |  |  |  |  |  |  |
| Mild | 48 | 28.7 | 1.22 | 0.0003 | - |  |  |  |  |  |  |  |  |
| Moderate | 74 | 30.5 | 1.53 | 0.382 | 0.369 | - |  |  |  |  |  |  |  |
| Significant | 28 | 29.5 | 0.785 | -0.460 | -0.451 | 0.791 |  |  |  |  |  |  |  |
| N-back Task | | | | | | | | | | | | | |
| Accuracy |  |  | 𝜒^2^(4) = 0.0831, $\eta$^2^ = -.0208 | | | | RT (ms) |  |  | 𝜒^2^(4) = 5.75, $\eta$^2^ = .009 | | | |
| No | 15 | 93.6 | - |  |  |  | No | 15 | 743 | - |  |  |  |
| Low | 49 | 94.6 | -0.218 | - |  |  | Low | 49 | 682 | -1.61 | - |  |  |
| Mild | 42 | 94.6 | -0.147 | 0.0957 | - |  | Mild | 42 | 661 | -2.34 | -1.09 | - |  |
| Moderate | 61 | 94.6 | -0.081 | 0.213 | 0.103 | - | Moderate | 61 | 685 | -1.66 | -0.030 | 1.11 | - |
| Significant | 26 | 94.8 | -0.194 | 0.0061 | -0.075 | -0.168 | Significant | 26 | 710 | -1.2 | 0.346 | 1.25 | 0.383 |
| RT variability (ms) | | | 𝜒^2^(4) = 4.12, $\eta$^2^ = .0007 | | | |  |  |  |  |  |  |  |
| No | 15 | 271 | - |  |  |  |  |  |  |  |  |  |  |
| Low | 49 | 239 | -1.89 | - |  |  |  |  |  |  |  |  |  |
| Mild | 42 | 243 | -1.59 | 0.372 | - |  |  |  |  |  |  |  |  |
| Moderate | 61 | 250 | -1.71 | 0.333 | -0.072 | - |  |  |  |  |  |  |  |
| Significant | 26 | 252 | -1.02 | 0.935 | 0.595 | 0.696 |  |  |  |  |  |  |  |
| Matrix Reasoning Task | | | | | | | | | | | | | |
| Matrix Reasoning (T-score) | | | 𝜒^2^(4) = 6.92, $\eta$^2^ = .014 | | |  |  |  |  |  |  |  |  |
| No | 16 | 51 | - |  |  |  |  |  |  |  |  |  |  |
| Low | 54 | 52 | 1.56 | - |  |  |  |  |  |  |  |  |  |
| Mild | 48 | 52 | 2.32 | 1.13 | - |  |  |  |  |  |  |  |  |
| Moderate | 74 | 53 | 1.66 | 0.0738 | -1.14 | - |  |  |  |  |  |  |  |
| Significant | 30 | 51.5 | 0.748 | -0.932 | -1.88 | -1.04 |  |  |  |  |  |  |  |

*Note.* RT: reaction time. The last three columns represent Dunn’s multiple comparison Z-test statistics. * *p* < .05, ** *p* < .01, *** *p* < .001.

**Table 5**

*Statistical Summary of Polynomial Trend Analysis.*

|  |  | Linear | | | Quadratic | | | Cubic | | | Quartic | | |
| --- | --- | --- | --- | --- | --- | --- | --- | --- | --- | --- | --- | --- | --- |
|  |  | $\hat{\beta}$ | SE | *p* | $\hat{\beta}$ | SE | *p* | $\hat{\beta}$ | SE | *p* | $\hat{\beta}$ | SE | *p* |
| Stroop |  |  |  |  |  |  |  |  |  |  |  |  |  |
| Accuracy | Congruent | -1.10 | 0.989 | 0.269 | -0.260 | 0.914 | 0.776 | -0.0392 | 0.722 | 0.957 | -0.740 | 0.669 | 0.270 |
|  | Incongruent | -0.638 | 0.757 | 0.401 | 0.163 | 0.700 | 0.816 | -0.101 | 0.553 | 0.855 | -0.492 | 0.512 | 0.338 |
|  | Difference | 0.459 | 0.708 | 0.518 | 0.423 | 0.655 | 0.519 | -0.0622 | 0.517 | 0.904 | 0.248 | 0.479 | 0.605 |
| Median reaction time | Congruent | 9.180 | 27.9 | 0.742 | 32.2 | 25.8 | 0.214 | 1.74 | 20.4 | 0.932 | 5.720 | 18.9 | 0.762 |
|  | Incongruent | -6.18 | 27.6 | 0.823 | 16.6 | 25.5 | 0.516 | -9.11 | 20.1 | 0.651 | 0.847 | 18.7 | 0.964 |
|  | Difference | -15.4 | 13.0 | 0.237 | -15.6 | 12.0 | 0.194 | -10.9 | 9.450 | 0.252 | -4.87 | 8.76 | 0.579 |
| Reaction time variability | Congruent | 88.8 | 35.5 | .0130* | 54.0 | 32.8 | 0.101 | 51.2 | 25.9 | .0490* | -8.06 | 24.0 | 0.737 |
|  | Incongruent | 114.5 | 53.5 | .0330* | 31.5 | 49.4 | 0.525 | 21.6 | 39.0 | 0.581 | -28.9 | 36.2 | 0.425 |
|  | Difference | 25.8 | 37.2 | 0.490 | -22.5 | 34.4 | 0.514 | -29.6 | 27.2 | 0.277 | -20.8 | 25.2 | 0.409 |
| N-back |  |  |  |  |  |  |  |  |  |  |  |  |  |
| Accuracy | | 2.38 | 1.88 | 0.207 | -0.693 | 1.75 | 0.692 | 0.232 | 1.400 | 0.868 | -0.916 | 1.310 | 0.485 |
| Median reaction time | | -27.2 | 27.8 | 0.329 | 57.9 | 25.8 | .026* | -16.7 | 20.7 | 0.419 | -13.7 | 19.4 | 0.481 |
| Reaction time variability | | -9.41 | 14.4 | 0.513 | 17.9 | 13.3 | 0.182 | -8.58 | 10.7 | 0.422 | 12.2 | 9.99 | 0.224 |
| MR T-score | | 1.03 | 1.72 | 0.548 | -4.27 | 1.59 | .008** | 0.0648 | 1.26 | 0.959 | 0.966 | 1.18 | 0.412 |

*Note.* $\hat{\beta}$: estimates, SE: standard error, *p*: *p*-value*. * p < .05, ** p < .01, *** p < .001*
